# Scenario Modeling of Urbanization Development and Water Scarcity Based on System Dynamics: A Case Study of Beijing–Tianjin–Hebei Urban Agglomeration, China

**DOI:** 10.3390/ijerph16203834

**Published:** 2019-10-11

**Authors:** Chao Bao, Dongmei He

**Affiliations:** 1 Institute of Geographic Sciences and Natural Resource Research, Chinese Academy of Sciences, Beijing 100101, China; hedongmei14@mails.ucas.ac.cn; 2 Key Laboratory of Regional Sustainable Development Modeling, Chinese Academy of Sciences, Beijing 100101, China; 3 College of Resources and Environment, University of Chinese Academy of Sciences, Beijing 100049, China

**Keywords:** urban agglomeration, healthy urbanization, water scarcity, water pollution, system dynamics, scenario simulation

## Abstract

Due to the accelerated process of urbanization in China, urban agglomerations have become the core areas for human settlement and economic development. High population and economic density has brought great pressure on water supply. Water scarcity is increasingly becoming one of the most important issues for the sustainable and healthy development of China’s urban agglomerations. In this paper, a system dynamics model was constructed to simulate the current conditions and future scenarios of urbanization development and water scarcity in the Beijing–Tianjin–Hebei (BTH) urban agglomeration in 2000–2030, by examining the interaction and feedback between the six major subsystems: water supply, water demand, water pollution, population urbanization, economic urbanization, and land urbanization. It is found that the South-to-North Water Diversion Project and the improved Reclaimed Water Reuse System may greatly increase the water supply. However, the speed of population urbanization and economic growth, the spatial structure of urban agglomeration and the water consumption pattern may determine the water demand. Although all scenarios may risk water scarcity in the future at some point, we could detect a comprehensive and relatively rational scenario to balance water scarcity, regional equity, and efficiency. It might help to synthetically understand the coordinated development mode between urbanization and water resources in Beijing–Tianjin–Hebei (BTH) urban agglomeration, and provide a useful analytical and decision support tool for scientists and policy-makers to achieve the sustainable urbanization development and water resource management.

## 1. Introduction

Water is a fundamental and indispensable resource for human survival and ecosystem evolution [1,2,3]. It also plays a supporting role in the sustainable development of the socio-economic system [4,5,6]. Due to rapid urbanization and industrialization, urban areas have undergone steady population growth and economic development. These areas face serious water scarcity and pollution both in developed and developing countries, which are the major threats to public health [7,8,9]. Specifically, urbanization could lead to water consumption increase in urban areas. It usually means more wastewater effluent and less water in rivers and lakes, both resulting in water quality deterioration [10,11]. Meanwhile, the increase in chemical concentrations due to water pollution could induce many diseases and deaths [12]. Therefore, urbanization, water scarcity, and water pollution are closely related, resulting in an important public health issue.

Regarding China, during the accelerated process of urbanization, urban agglomerations have become the core areas for human settlement and economic development [13,14]. High population and economic density has brought water scarcity, water pollution, and related eco-environmental issues, becoming a key factor to constraining socio-economic development [14,15]. The conflicts between water scarcity and urbanization development have aroused concern worldwide [16,17]. Previous studies have focused on the relationship between urbanization and water resources, e.g., the positive and negative effects of urbanization on water resources [18,19,20,21], the quantitative relationship between population urbanization ratio and total water consumption [22,23,24,25], and the coupling and decoupling relationship between urbanization quality and water scarcity [5]. However, the interactions between urbanization and water resources are very complicated, and the relationships between urbanization and water resources have different forms in different stages and regions [26,27,28]. To understand the inherent mechanism and the potential consequences of decisions, it is necessary to simulate the complex interactions and dynamic feedbacks between the natural and socio-economic factors of urbanization and water resources, particularly in China’s fast-growing urban agglomerations.

The system dynamics (SD) model, developed by Forrester [29,30,31], is a computer-based method grounded in the modern theory of nonlinear dynamics and the feedback control theory [32,33]. It has the advantage to model system feedbacks, amplifications, behavioral responses, structural relationships, nonlinearities, time delays, and alternative future scenarios [34]. In the past few decades, the SD model has been widely applied in sustainability to facilitate a holistic analysis of complex human–environmental systems [35,36]. Among them, human–water systems in various spatial scales such as countries, regions, cities, river basins or watersheds have formed a large amount of literatures [37,38,39,40,41,42,43,44]. The diversity of SD model applications has improved understanding of the complex interactions between human–water systems. However, there is still a need for SD models that adequately integrate various physical, social, and economic factors that determine the current and future dynamics of human–water systems [41,42]. Moreover, most previous studies only focused on one entire country, region, city, or river basin [42,43]. Few studies focused on the impacts of the spatial structure of the research unit, or the interactions between intra regions or cities. Meanwhile, few studies focused on the impacts of urbanization, or the urban–rural structure of population and economy, on water demand. Besides, the impacts of water pollution were often ignored when quantitatively modelling the relationship between urbanization and water scarcity [44]. As a consequence, the SD model approaches in urban agglomeration are scarce, and there is limited knowledge and understanding about the interaction and feedback behaviors of human–water systems in urban agglomeration.

Therefore, to contribute in filling the above literature gaps, we construct a SD model to simulate the current conditions and future scenarios of urbanization development and water scarcity in Beijing–Tianjin–Hebei (BTH) urban agglomeration. It not only considers the impacts of water pollution and the urban–rural structure of population and economy on water resources scarcity, but also takes the spatial structure of urban agglomeration into consideration. The effects from water consumption patterns of the residents, agriculture and industry are also evaluated in this SD model. The two main objectives of this paper are (1) to assess the applicability of SD model for evaluating how urbanization development affect water scarcity in urban agglomeration, and (2) to analyze the current situation and future trends of the urban agglomeration development path and obtain a sustainable utilization pattern of water resources. By this useful analytical and decision support tool, it might help to synthetically understand the coordinated development mode among water supply, water demand, water pollution, population urbanization, economic urbanization, and land urbanization in BTH urban agglomeration, and promote sustainable urbanization development, water resource management, and public health in similar regions.

## 2. Materials and Methods

### 2.1. Study Area and Data Sources

**Figure 1:**
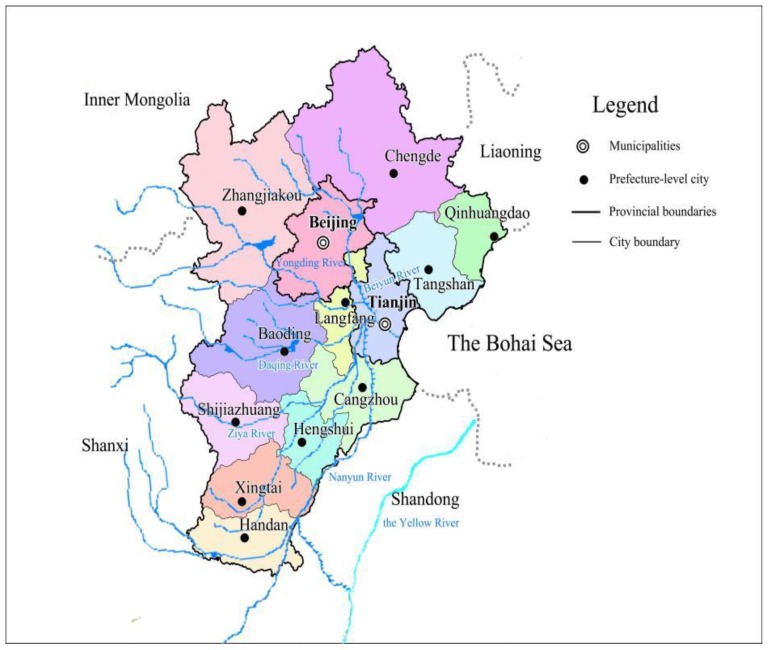
Sketch map of Beijing–Tianjin–Hebei (BTH) urban agglomeration.

BTH urban agglomeration, one of the world’s largest agglomerations, is located in the Haihe River Basin and the North China Plain. It has 13 cities at prefecture level and above, including Beijing, Tianjin, Shijiazhuang, Tangshan, Qinhuangdao, Handan, Xingtai, Baoding, Zhangjiakou, Chengde, Cangzhou, Langfang, and Hengshui (Figure 1). It covers an area of 0.2168 million km^2^, which accounts for 2.26% of China. It belongs to a typical continental monsoon climate type, which is characterized by hot and rainy summers, and cold and dry winters. As a semi-humid and semi-arid region, the mean annual precipitation varies from 400 to 800 mm, and the mean annual potential evaporation ranges from 1100 to 2000 mm [45,46]. According to statistics, in 2014, it has a population of 112 million, which accounts for 8.18% of China. The urbanization rate, which is the ratio of urban population to total population, is 61.0%. The gross domestic product (GDP) is 6649 billion yuan at current price, which accounts for 10.45% of China. It is the political, economic and cultural center of China, as well as one of the most competitive support platforms for China’s international economic system [47,48,49]. However, the gross amount of water resources is 20.369 billion m^3^, only 0.75% of China. Its per capita water resources are 182 m^3^, only 9.14% of the national average and much lower than 500 m^3^, meaning an extremely severe water scarcity [6]. The total water consumption is 25 billion m^3^, accounting for 4.10% of China. Its per capita water consumption is 224 m^3^, which is 50% of the national average. Water consumption per ten thousand yuan GDP is 38 m^3^, which is 40% of the national average, meaning high water use efficiency [45]. In a word, BTH urban agglomeration is not only one of the areas owning the highest population density and the most vigorous economics, but also one of the most prominent regions of contradiction between human and water in China and even throughout the world [50,51].

The basic data in this paper include socio-economic data, water resources data, land use data and eco-environmental data for the 13 cities at prefecture level and above in 2000–2014. The socio-economic data, land use data, and eco-environmental data are all obtained from the past years statistical yearbooks of Beijing, Tianjin, and Hebei, respectively. The water resources data are all obtained in the past years water resources communique of Beijing, Tianjin, and Hebei respectively. To make economic data comparable in time series, all the economic data are calculated at comparable prices in 2000. For the total and average indexes of the whole region of BTH urban agglomeration, we firstly sum up the total indexes of the 13 cities at prefecture level and above, and then calculate the average indexes.

### 2.2. General Framework and Concept Model

**Figure 2:**
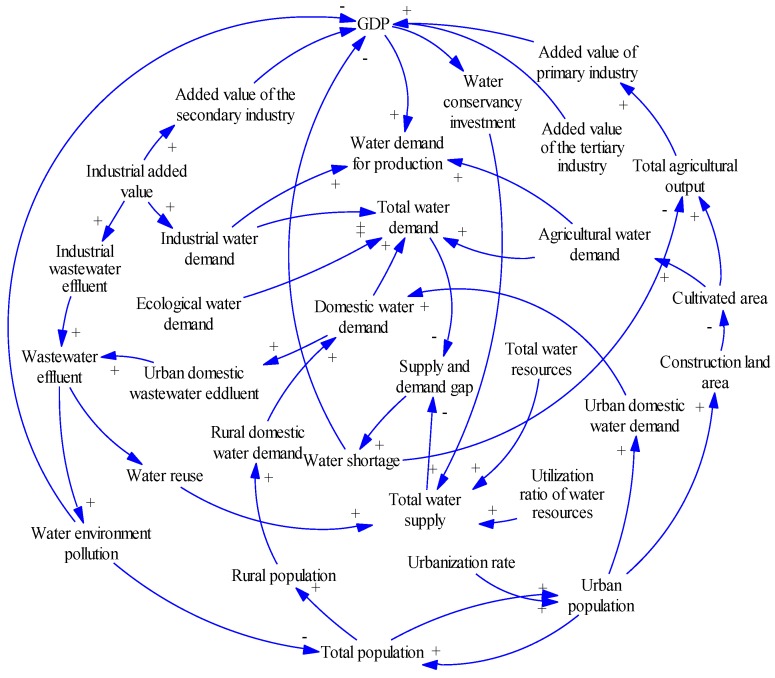
Causal loop diagram of water resources and urbanization development. Direct influences of key variables are shown as solid arrows; ‘+’ indicates a positive link; ‘−’ indicates a negative link.

A system dynamics model starts with the development of a dynamic hypothesis, generally referred to a causal loop diagram, which is a useful qualitative analytical tool for representing the relationships among system variables that produce dynamic feedback structure [32,41]. We use Vensim 5.11 software (Ventana Systems Inc., http://www.ventanasystems.com/) to draw the causal loop diagram of BTH urban agglomeration to examine the feedback processes between water resource and urbanization development (Figure 2).

As shown in Figure 2, the positive (+) or negative (−) impact of a variable on another variable is indicated by the solid arrow. The variables are directly connected to each other by links, generating positive and negative feedback loops. The positive feedback loops indicate a reinforcing change within the water resources and urbanization development system. The negative feedback loops indicate a depressed and balancing process. In this concept model, the main loops of cause and effect are as follows.

(1) A negative feedback loop between population and water demand: Total population →+ Domestic water demand →+ Total water demand →+ Water supply and demand gap →- Total population.

(2) A negative feedback loop between population and water environment: Total population→+ Urban domestic water demand →+ Wastewater effluent →+ Water environment pollution→- Total population.

(3) A negative feedback loop between economy and water demand: GDP →+ Water demand for production →+ Total water demand →+ Water supply and demand gap →- GDP.

(4) A negative feedback loop between economy and water environment: GDP →+ Water demand for production →+ Total water demand →+ Wastewater effluent →+ Water environmental pollution →- GDP.

(5) A positive feedback loop between economy and water demand: GDP →+ Water conservancy investment →+ Total water supply →- Water supply and demand gap →+ GDP.

The above negative feedback loops indicate that population growth and economic development may increase water demand, but water scarcity and water pollution may restrain the population growth and economic development. The above positive feedback loops indicate that economic development may increase water supply by increasing investment in water conservancy, and lessen the constraints caused by water scarcity and water pollution. Besides the above-mentioned main feedback loops, there are other complex interactions between water resources and urbanization development system through its structure and efficiency changes (Figure 2).

### 2.3. Dynamic Simulation Model Settings and Description

**Figure 3:**
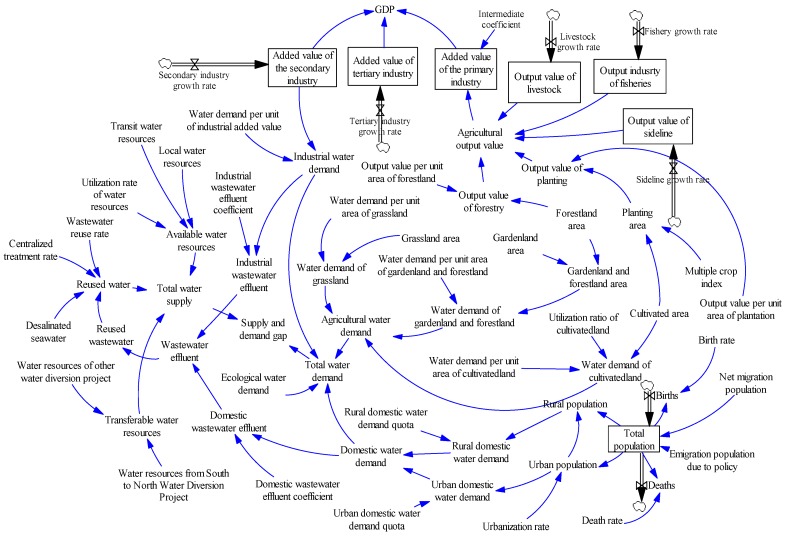
Stock and flow diagram of system dynamics model of urbanization development and water scarcity.

Based on the causal loop diagram which is conceptual and qualitative, we divide the water resources and urbanization development system into six major subsystems: water supply, water demand, water pollution, population urbanization, economic urbanization, and land urbanization. Subsequently, we use Vensim 5.11 software to draw a stock and flow diagram (Figure 3), which can quantificationally simulate system behavior for each city of BTH urban agglomeration over a 30-year period (2000–2030). We consider not only the interactions among the stocks (level variables) and flows (rate variables) of one city’s water resources and urbanization development system, but also the impacts of the water resources and population flows among its neighboring cities. Through the scenario simulations of the 13 cities at prefecture level and above, we sum the total indexes for the whole region of BTH urban agglomeration, and reveal the effects of the spatial structure and the urban–rural structure changes on water scarcity.

#### 2.3.1. Water Supply Subsystem

The core variable of water supply subsystem is total water supply (*TWS*), which is composed of local water resources (*LWR*), transit water resources (*TWR*), utilization rate of water resources (*RT*), transfer water resources (*TFWR*), and unconventional water resources (*UWR*). The description function is denoted by Equation (1):

(1) $$TWS_{t}=(LWR_{t}+TWR_{t})\times RT_{t}+TFWR_{t}+UWR_{t}$$

where *TFWR_t_* is the water supply by the South-to-North Diversion Project at time *t*. *UWR_t_* contains the desalinated seawater (*DSW*) and reused wastewater (*RWW*) at time *t*.

#### 2.3.2. Water Demand Subsystem

The core variable of water demand subsystem is total water demand (*TWD*), which is composed of agricultural water demand (*AWD*), industrial water demand (*IWD*), domestic water demand (*DWD*), and ecological water demand (*EWD*). The description function is denoted by Equation (2):

(2) $$TWD_{t}=AWD_{t}+IWD_{t}+DWD_{t}+EWD_{t}$$

Agricultural water demand (*AWD*) is composed of water demand for farmland (*FWD*), woodland (*WWD*), orchard (*OWD*), and grassland (*GWD*). Each kind of water demand is the product of land use area (*A_i_*), the corresponding coefficient of irrigation (*α_i_*), and the irrigation water-demand quota (*β_i_*). Thus, the water demand subsystem is connected to the land urbanization subsystem.

(3) $$AWD_{t}=FWD_{t}+WWD_{t}+OWD_{t}+GWD_{t}=\sum_{i=1}^{4}\left(A_{it}\times \alpha_{it}\times \beta_{it}\right)$$

Industrial water demand (*IWD*) is the product of industrial added value (*IAV_t_*) and industrial water-demand quota, which is defined as water demand per unit of industrial added value (*IWQ_t_*). Thus, the water demand subsystem is connected to the economic urbanization subsystem.

(4) $$IWD_{t}=IAV_{t}\times IWQ_{t}$$

Domestic water demand (*DWD*) is composed of urban domestic water demand (*UDWD*) and rural domestic water demand (*RDWD*), which is the product of urban (*UP*) and rural population (*RP*) and the corresponding water-demand quota (*UPQ* and *RPQ*), respectively. Thus, the water demand subsystem is connected to the population urbanization subsystem.

(5) $$DWD_{t}=UDWD_{t}+RDWD_{t}=UP_{t}\times UPQ_{t}+RP_{t}\times RPQ_{t}$$

Ecological water is mainly used for irrigation in urban green space and water discharge to rivers or lakes for sustaining the functionality of freshwater ecosystems. To achieve sustainable development, we calculate ecological water demand (*EWD*) as Equation (6):

(6) $$EWD_{t}=\int_{t_{0}}^{t}r_{e}dt+EWD_{t_{0}}$$

where *t* is the current time, *t*_0_ is the initial time, and *r_e_* is the growth of *EWD* at time *t*.

#### 2.3.3. Water Pollution Subsystem

The core variable of water pollution subsystem is wastewater effluent (*WWE*). It is composed of industrial wastewater effluent (*IWWE*) and domestic wastewater effluent (*DWWE*), which is the product of industrial (*IWD*) and domestic water demand (*DWD*) and the corresponding effluent coefficient (*γ* and *δ*), respectively. Thus, the water pollution subsystem is connected to the water demand subsystem.

(7) $$WWE_{t}=IWWE_{t}+DWWE_{t}=IWD_{t}\times \gamma_{t}+DWD_{t}\times \delta_{t}$$

As reused wastewater (*RWW*) is the product of wastewater effluent (*WWE*) and wastewater reuse rate (*WWRR*), the water pollution subsystem is also connected to the water supply subsystem.

(8) $$RWW_{t}=WWE_{t}\times WWRR_{t}$$

#### 2.3.4. Population Urbanization Subsystem

The core variables of population urbanization subsystem are total population (*P*), urban population (*UP*) and rural population (*RP*), which are direct or indirect factors that affect the water demand and supply as mentioned above. The description functions are mainly as follows:

(9) $$P_{t}=\int_{{}_{t0}}^{t}\left[Birth(t)-Death(t)\right]dt+\int_{{}_{t0}}^{t}\left[InP(t)-OutP(t)\right]dt+P_{t_{0}}$$

(10) $$UP_{t}=P_{t}\times \mu_{t}$$

(11) $$RP_{t}=P_{t}-UP_{t}$$

where *P_t_* is population in the current time; *P*_*t*0_ is population in the initial time; *Birth(t)* and *Death(t)* are the births and deaths at time *t*, respectively; *InP(t)* and *OutP(t)* are the immigration and emigration at time *t*, respectively; and *μ_t_* is population urbanization ratio.

#### 2.3.5. Economic Urbanization Subsystem

The core variable of economic urbanization subsystem is gross domestic product (*GDP*), which not only determines the water demand, but also affects the water supply through water conservancy investment (*WCI*). It is composed of added value of the primary industry (*AVPI*), added value of the secondary industry (*AVSI*), and added value of the tertiary industry (*AVTI*).

(12) $$GDP_{t}=AVPI_{t}+AVSI_{t}+AVTI_{t}$$

The added value of the primary industry (*AVPI*) is the product of agricultural output value (*AOV*) and the corresponding coefficient (*η*). The *AOV* is composed of output value of planting (*OVP*), forestry (*OVF*), livestock (*OVL*), fishery (*OVFY*), and sideline (*OVS*), which is calculated by growth rate method and connected to agricultural water demand (*AWD*) and different kinds of land use area (*A_i_*), respectively.

(13) $$AVPI_{t}=AOV_{t}\times \eta_{t}=(OVP_{t}+OVF_{t}+OVL_{t}+OVFY_{t}+OVS_{t})\times \eta_{t}$$

The added value of the secondary industry (*AVSI*) is the product of industrial added value (*IAV*) and the corresponding coefficient (*θ*). The *IAV* is calculated by growth rate method and connected to industrial water demand (*IWD*) as mentioned above.

(14) $$AVSI_{t}=IAV_{t}\times \theta_{t}$$

The added value of the tertiary industry (*AVTI*) is calculated by growth rate method as Equation (15):

(15) $$AVTI_{t}=\int_{t_{0}}^{t}R_{T}dt+AVTI_{t_{0}}$$

where *t* is the current time, *t*_0_ is the initial time, and *RT* is the growth of *AVTI* at time *t*.

#### 2.3.6. Land Urbanization Subsystem

The core variables of land urbanization subsystem are farmland area (*A_farm_*), woodland area (*A_wood_*), orchard (*A_orch_*), grassland (*A_grass_*), and construction land area (*A_con_*). The description functions are mainly as follows:

(16) $$A_{it}=\int_{{}_{t0}}^{t}\left[AInc_{i}(t)-ADcr_{i}(t)\right]dt+A_{it_{0}}$$

where *A_it_* is the *i*th kind of land use area in the current time; *A_it0_* is the *i*th kind of land use area in the initial time; and *AInc_i_(t)* and *ADcr_i_(t)* are the increase and decrease of the *i*th kind of land use area at time *t*, respectively. As mentioned above, they are connected to the water demand subsystem and the economic urbanization subsystem.

### 2.4. Parameters and Scenario Settings

The objective of the SD model is to provide a decision support tool for policy-makers to explore plausible policy scenarios and grasp the sustainable water resources management. Due to the complexity of the water resources and urbanization development system, it is difficult to determine how it works. To overcome this difficulty, for general parameters, we assume that their changes are consistent with their respective historical development trends during 2000–2014. However, for key parameters, we design some scenarios from three major aspects to simplify the simulations.

#### 2.4.1. Scenario Design of Water Supply

Water supply is determined by complex natural factors and human activities [2,3]. Parameters such as transfer water resources (*TFWR*), the utilization rates of water resources (*RT*), the industrial and domestic wastewater effluent coefficient (*γ* and *δ*), the desalinated seawater (*DSW*), and wastewater reuse rate (*WWRR*), are modeled by numerical experiments according to the historical data during 2000–2014, or by table functions according to the Government’s planning. For parameters such as local water resources (*LWR*) and transit water resources (*TWR*), we use their average annual values during 2000–2014 as a baseline. However, local water resources and transit water resources in BTH urban agglomeration have greatly decreased since 1956 [45,46]. Moreover, the actual water withdrawal of the South-to-North Water Diversion Project is usually lower than the planning due to engineering and financial situation. Therefore, we assume three scenarios for water supply:

(1) High water supply scheme, in which local water resources (*LWR*) and transit water resources (*TWR*) are 100% of the average annual values during 2000–2014, and transfer water resources (*TFWR*) are 100% of the planning values.

(2) Medium water supply scheme, in which local water resources (*LWR*) and transit water resources (*TWR*) are 75% of the average annual values during 2000–2014, and transfer water resources (*TFWR*) are 75% of the planning values.

(3) Low-water supply scheme, in which local water resources (*LWR*) and transit water resources (*TWR*) are 50% of the average annual values during 2000–2014, and transfer water resources (*TFWR*) are 50% of the planning values.

#### 2.4.2. Scenario Design of Water Consumption Mode

Water-demand quotas serve as the control variables of water consumption mode. They are affected by complex factors such as local policies, regulations, water prices, living standards, technologies, public awareness of water conservation, and water resources management [37,38,43]. To simulate them succinctly and rationally, we assume two kinds of water consumption mode:

(1) Water-consuming mode, in which all the water-demand quotas are forecasted by the trend extrapolation of the historical data during 2000–2014, and the results are then used as a baseline.

(2) Water-saving mode, in which all the water-demand quotas are less than the baseline according to the possibility of water saving in each city. For instance, the irrigation water-demand quotas for farmland, woodland, orchard, and grassland are 5–10 m^3^/ha less than the baseline. The industrial water-demand quotas are 1–2 m^3^/ ten thousand yuan less than the baseline. The urban and rural domestic water-demand quotas are 5–10 L per capita per day less than the baseline. On the whole, through the water-saving measures scenario and no water-saving measures scenario, we may detect the impacts of water consumption mode on the changes of water demand in BTH urban agglomeration.

#### 2.4.3. Scenario Design of Urbanization Development

The socio-economic development system, including the population urbanization subsystem, economic urbanization subsystem, and land urbanization subsystem, is affected by various policies and uncertain factors. We may set numerous scenarios for it. To be concise and grasp the focal point, we assume three kinds of urbanization development modes:

(1) Core development mode, in which BTH urban agglomeration continues the previous development mode. The growth rates of population and economy of all cities are forecasted by the trend extrapolation of the historical data during 2000–2014, and the results are then used as a baseline. Beijing is continuing to be the core city to gather more population and industries due to its agglomeration effects. It has relatively high growth rates of population and economy.

(2) Subcore development mode, in which the population and urban function of Beijing are relieved. Its population urbanization ratio is one percentage point lower than its baseline, and its growth rate of each industry is 0.5 percentage point lower than its baseline. Tianjin, Shijiazhuang, Baoding, Tangshan, Langfang, and Cangzhou are considered as subcores and key development areas. Their population urbanization ratios are one percentage point higher than the baseline, and their growth rates of each industry are 0.5 percentage point higher than the baseline, respectively.

(3) Multinode development mode, in which other cities such as Qinhuangdao, Handan, Xingtai, Hengshui, Chengde, and Zhangjiakou are considered as key development areas to pursue a balanced development in BTH urban agglomeration. Their population urbanization ratios are one percentage point higher than the baseline, and their growth rates of each industry are 0.5 percentage points higher than the baseline, respectively.

## 3. Results and Discussion

### 3.1. Validation Results of the SD Model

**Figure 4:**
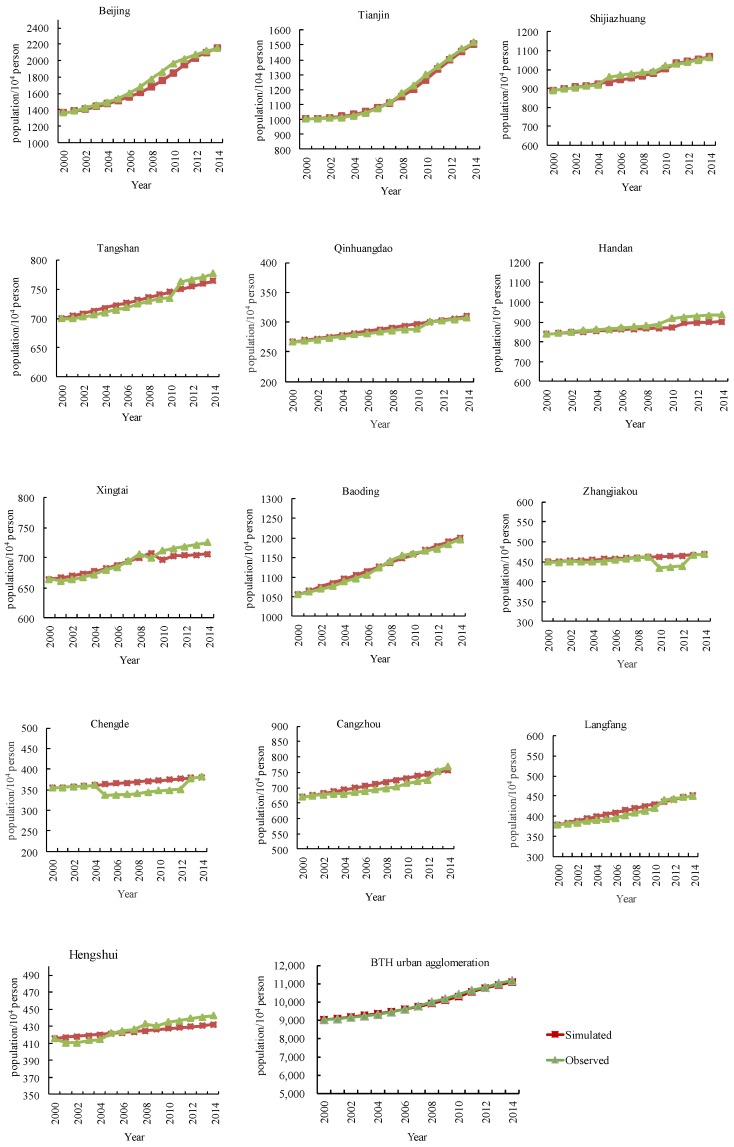
The comparison between observed and simulated results of population in 13 cities in the Beijing–Tianjin–Hebei (BTH) urban agglomeration.

**Figure 5:**
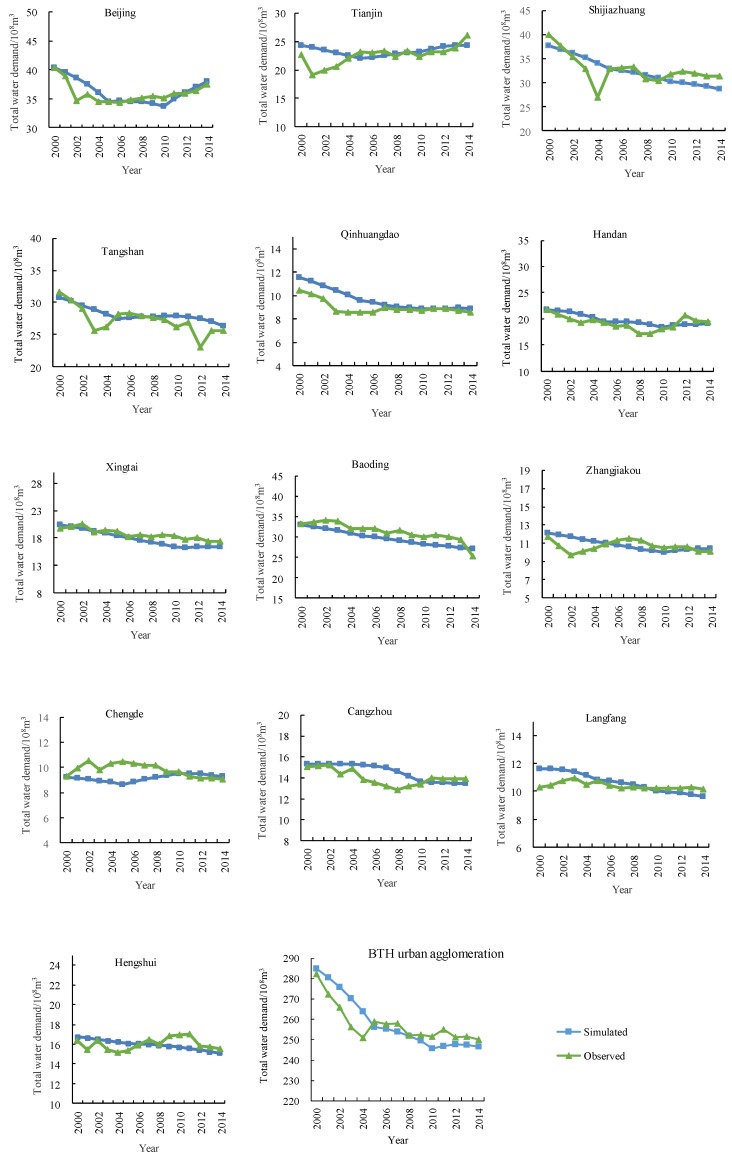
The comparison between observed and simulated results of total water demand in 13 cities in the Beijing–Tianjin–Hebei (BTH) urban agglomeration.

The validity of the SD model is the precondition for forecasting and analyzing the future scenarios of urbanization development and water scarcity in BTH urban agglomeration. It could be indicated by the errors between the simulated results and the existing historical data. If the errors between the simulated and true values are in the interval −10% to 10%, the results could be acceptable. However, if the errors are larger, the model must be modified. Therefore, we select some important variables to test the SD model, including total population (*P*), urban population (*UP*), gross domestic product (*GDP*), added value of the secondary industry (*AVSI*), added value of the tertiary industry (*AVTI*), farmland area (*A_farm_*), construction land area (*A_con_*), total water demand (*TWD*), agricultural water demand (*AWD*), industrial water demand (*IWD*), domestic water demand (*DWD*), wastewater effluent (*WWE*), industrial wastewater effluent (*IWWE*), and domestic wastewater effluent (*DWWE*). The absolute values of the average errors of these variables during 2000–2014 in all cities are less than 10%, and most of them are less than 5%. For instance, Figure 4 and Figure 5 show the comparison between the observed and simulated results of total population (*P*) and total water demand (*TWD*) in 13 cities in BTH urban agglomeration during 2000–2014, respectively. The trends of the simulated values are generally consistent with the observed data in all cities during 2000–2014. It indicates that the simulated results reflect reality well, and thus the SD model is well calibrated and could effectively reflect the situation of the actual system in BTH urban agglomeration.

### 3.2. Water Supply under Different Scenarios

As the South-to-North Water Diversion Project is taking effect step by step, water supply planning for Beijing, Tianjin, and Hebei will increase 12 × 10^8^ m^3^, 15 × 10^8^ m^3^, and 30 × 10^8^ m^3^ in 2030, respectively. Water supply planning for each city in the south-central of Hebei province will increase ~3 × 10^8^ m^3^. Thus total water supply in BTH urban agglomeration under different scenarios will be increased by 28.5 × 10^8^ m^3^ to 57 × 10^8^ m^3^. On the other hand, the wastewater reuse rate in BTH urban agglomeration will increase from 21%, in 2015, to ~36%, in 2030. Specifically, Beijing will increase from 60% in 2015 to ~70% in 2030. Tianjin will increase from 30% in 2015 to ~50% in 2030. Most cities in Hebei province will reach 15% to 30% in 2030. Thus total reused wastewater in BTH urban agglomeration will be ~50 × 10^8^ m^3^ to 90 × 10^8^ m^3^. The South-to-North Water Diversion Project and the improved Reclaimed Water Reuse System will greatly increase water supply. On the whole, in the high water supply scheme, total water supply in BTH urban agglomeration will increase from 265.8 × 10^8^ m^3^ in 2015 to 317.7 × 10^8^ m^3^ in 2030. In the medium water supply scheme, it will increase from 248.6 × 10^8^ m^3^ in 2015 to 286.8 × 10^8^ m^3^ in 2030. In the low-water supply scheme, it will increase from 193.3 × 10^8^ m^3^ in 2015 to 245.0 × 10^8^ m^3^ in 2030. Specific situation is listed in Table 1.

### 3.3. Urbanization Development under Different Scenarios

In the core development mode, total population in BTH urban agglomeration will increase from 118.9 million in 2020 to 132.6 million in 2030. The urbanization rate will increase from 67.5% in 2020 to 75.0% in 2030. The gross domestic product (*GDP*) will increase from 7707 billion yuan in 2020 to 15,371 billion yuan in 2030. In the subcore development mode, total population in BTH urban agglomeration will increase from 118.9 million in 2020 to 132.8 million in 2030. The urbanization rate will increase from 68.6% in 2020 to 75.7% in 2030. The gross domestic product (*GDP*) will increase from 7769 billion yuan in 2020 to 15,986 billion yuan in 2030. In the multinode development mode, total population in BTH urban agglomeration will increase from 118.9 million in 2020 to 133.6 million in 2030. The urbanization rate will increase from 67.8% in 2020 to 74.2% in 2030. The gross domestic product (*GDP*) will increase from 7752 billion yuan in 2020 to 15,255 billion yuan in 2030. Among the three kinds of scenarios, the subcore development mode has the largest urbanization rate and economic growth, whereas the multinode development mode has the lowest urbanization rate and economic growth. On the whole, the subcore development mode strikes a balance between development efficiency and regional equity while the other two are both one-sided. Great difference can be seen from the 13 cities in BTH urban agglomeration (Table 2).

### 3.4. Water Scarcity under Different Scenarios

**Figure 6:**
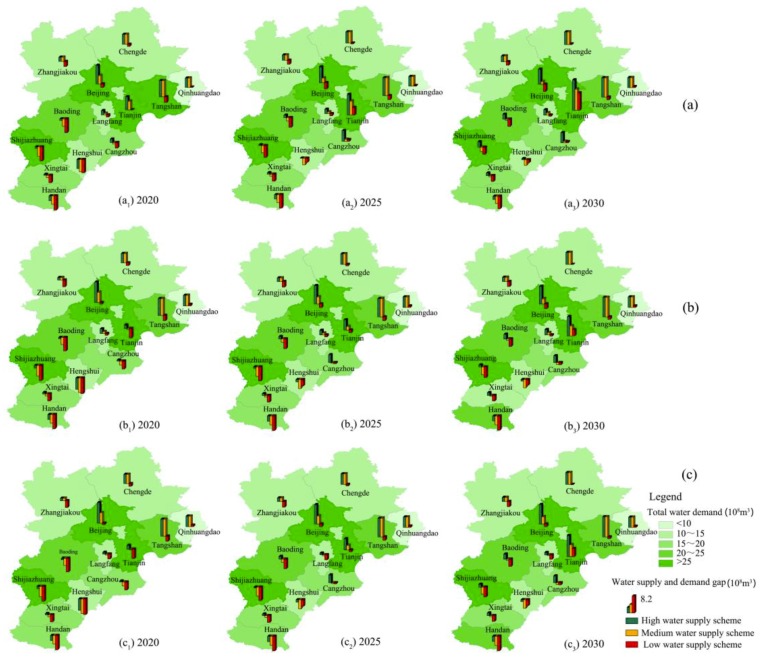
Water supply and demand gap in water-consuming mode under different urbanization development scenarios in BTH urban agglomeration. (**a**) Core development mode. (**b**) Subcore development mode. (**c**) Multinode development mode.

**Figure 7:**
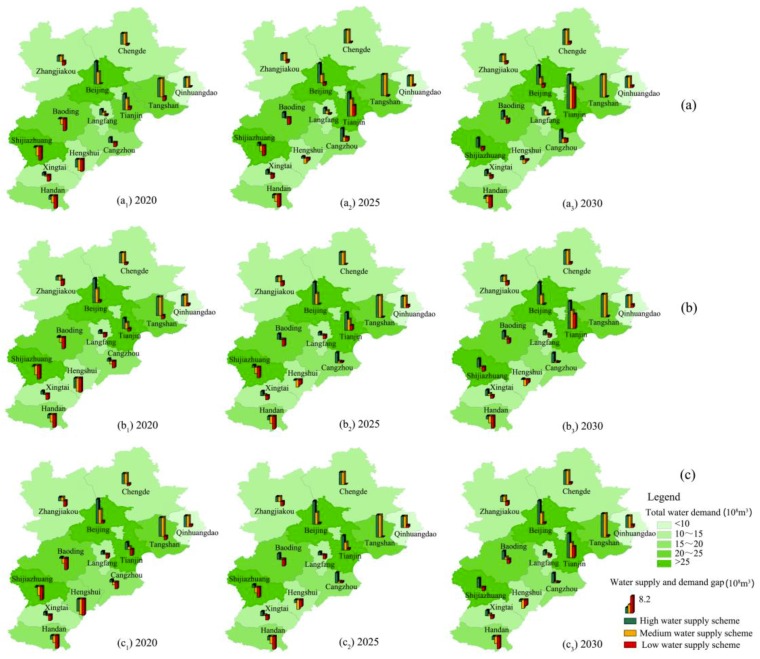
Water supply and demand gap in water-saving mode under different urbanization development scenarios in BTH urban agglomeration. (**a**) Core development mode. (**b**) Subcore development mode. (**c**) Multinode development mode.

As shown in Figure 6 and Figure 7, water scarcities in water-consuming and water-saving modes under different urbanization development scenarios in BTH urban agglomeration are also different. Almost all scenarios have risks of water shortage for the 13 cities as at least one pillar grows downward in Figure 6 and Figure 7, which means that the water supply and demand gap is negative. However, the water shortage ratios and water use efficiencies are various under different scenarios. From them, we could choose a comprehensive and relatively rational scenario to balance water scarcity, regional equity, and efficiency.

#### 3.4.1. Scenarios of Water-Consuming Mode

If the water-demand quotas develop in accordance with historical trends, total water demand in 2030 in BTH urban agglomeration will be 259.6 × 10^8^ m^3^, 269.8 × 10^8^ m^3^, and 269.4 × 10^8^ m^3^ in the core, subcore, and multinode development modes, respectively. It is the lowest in the core development mode, and they are about the same in the subcore and multinode development mode. Water use efficiency will be 592.2 yuan/m^3^, 592.4 yuan/m^3^, and 566.2 yuan/m^3^ correspondingly. It is the lowest in the multinode development mode, and they are about the same in the core and subcore development mode. It is seen that the core and subcore development mode may save more water and ease water scarcity due to higher water use efficiency. However, they may increase regional disparities and injustice according to the distribution of *GDP* (Table 2).

Moreover, in the low-water supply scheme, the water supply and demand gap in 2030 in BTH urban agglomeration will be −14.5 × 10^8^ m^3^, −24.8 × 10^8^ m^3^, and −24.4 × 10^8^ m^3^ in the core, subcore, and multinode development modes, respectively. The pillars in most cities grow downward in Figure 6c. It indicates that if BTH urban agglomeration does not make full use of the South-to-North Water Diversion Project and the improved Reclaimed Water Reuse System; it may still face water scarcity in the future 15 years no matter what kind of development mode it takes.

Finally, even in the high and medium water supply scheme, though the water supply and demand gaps of the whole urban agglomeration are above zero, those of some cities are still negative and the absolute values are relatively large (Figure 6a,b). It indicates that water deficiency in local regions will exist for a long time in BTH urban agglomeration unless extreme measures are taken to limit the development of these regions. Therefore, to reduce the risk of water shortage, a more severe water-saving mode had better be considered.

#### 3.4.2. Scenarios of Water-Saving Mode

If the water-demand quotas are reduced according to the possibility of water saving in each city, total water demand in 2030 in BTH urban agglomeration will be 244.7 × 10^8^ m^3^, 251.6 × 10^8^ m^3^, and 253.0 × 10^8^ m^3^ in the core, subcore, and multinode development modes, respectively. Compared with the water-consuming mode, it may save 14.8 × 10^8^ m^3^, 18.3 × 10^8^ m^3^, and 16.4 × 10^8^ m^3^ of water resources. Correspondingly, water use efficiency will be 628.0 yuan/m^3^, 635.5 yuan/m^3^, and 602.9 yuan/m^3^. It is seen that water-saving potential and water use efficiency are both the highest in the subcore development mode. In other words, the subcore development mode could give due consideration to efficiency and fairness, as well as make full use of its advantages. However, it is undesirable to focus only on one city or equally on all cities.

Specifically, in the low water supply scheme, the water supply and demand gap in 2030 in the whole urban agglomeration will be 0.3 × 10^8^ m^3^, −6.5 × 10^8^ m^3^, and −8.0 × 10^8^ m^3^ in the core, subcore, and multinode development modes, respectively. The risks of water scarcity in the future 15 years may gradually decrease to a low level. Although the water supply and demand gaps in some of the 13 cities are still negative (Figure 7c), the water shortage ratios are mostly below 10%. It indicates that water-saving is also one of the important keys to reduce water scarcity for BTH urban agglomeration. As long as BTH urban agglomeration adopted strict water-saving measures, the risks of water scarcity could be controlled at a safety level.

Besides, in the high water supply scheme, the water supply and demand gap in 2030 in the whole urban agglomeration will be 72.9 × 10^8^ m^3^, 66.1 × 10^8^ m^3^, and 64.6 × 10^8^ m^3^ in the core, subcore, and multinode development modes, respectively. In the medium water supply scheme, they will be 42.1 × 10^8^ m^3^, 35.3 × 10^8^ m^3^, and 33.8 × 10^8^ m^3^, respectively. Water supplies of most cities are larger than water demands as the pillars grow upward in Figure 7a,b. It indicates that, if BTH urban agglomeration adopts strict water-saving measures and makes full use of various water resources, there will be surplus water resources to restore the degraded ecosystem.

## 4. Conclusions

This paper constructed a SD model to simulate the current conditions and future scenarios of urbanization development and water scarcity in BTH urban agglomeration in 2000–2030, considering the nexus of water supply, water demand, water pollution, population urbanization, economic urbanization, and land urbanization, which are all closely related to public health. Based on the analysis and comparison of various scenarios, a relatively rational scheme to balance water scarcity and urbanization development was explored. The following conclusions were obtained.

(1) In the future 15 years, total water supply in BTH urban agglomeration has great uncertainty due to climate change, decrease of upstream water inflow, increase of water transfer, and utilization of unconventional water resources. It ranges from 245.0 × 10^8^ m^3^ to 317.7 × 10^8^ m^3^ in 2030 under different scenarios. The South-to-North Water Diversion Project and the improved Reclaimed Water Reuse System may greatly increase the water supply. Therefore, it is necessary to make full use of them to guarantee a steady water supply.

(2) The speed of population urbanization and economic growth, the spatial structure of urban agglomeration and the water consumption pattern may determine the water demand. All scenarios may have risks of water scarcity for the 13 cities, especially in the low-water supply scheme. However, if the local governments adjust the urbanization development and water consumption mode, and adopt vigorous measures to support a high and medium water supply, the water shortage ratios of the 13 cities may gradually decrease to a safety level. Some surplus water resources may be used to restore the degraded ecosystem.

(3) Among different scenarios, water-saving potential and water use efficiency in the water-saving and subcore development modes are the highest. It may be chosen as a comprehensive and relatively rational scenario to balance water scarcity, regional equity, and efficiency. In this scenario, total population in BTH urban agglomeration will increase from 118. 9 million, in 2020, to 132.8 million, in 2030. The urbanization rate will increase from 68.6% in 2020 to 75.7% in 2030. GDP will increase from 7769 billion yuan in 2020 to 15,986 billion yuan in 2030. The whole region will be not short of water in the high and medium water supply scheme. Even in the low-water supply scheme, the water shortage ratio will decrease from 15.0% in 2020 to 2.6% in 2030. However, water-saving potential and water use efficiency in the water-consuming and multinode development modes are the lowest on the whole, suggesting that high water consumption and completely balanced development strategy should be avoided.

## Figures and Tables

**Table 1:** Water supply of different schemes in BTH urban agglomeration (10^8^ m^3^).

Cities	High Water Supply Scheme				Medium Water Supply Scheme				Low-Water Supply Scheme			
	2015	2020	2025	2030	2015	2020	2025	2030	2015	2020	2025	2030
Beijing	43.8	50.8	52.8	55.1	41.8	45.8	47.3	49.1	33.1	40.0	42.0	44.3
Tianjin	21.4	31.5	38.0	45.4	21.4	29.3	34.7	40.9	15.1	25.2	31.7	39.1
Shijiazhuang	26.8	27.1	27.6	29.8	22.9	23.2	23.7	25.1	20.7	21.1	21.5	23.7
Tangshan	32.7	33.0	33.3	33.6	32.7	33.0	33.3	33.6	22.0	22.3	22.6	22.9
Qinhuangdao	13.2	13.3	13.3	13.4	13.2	13.3	13.3	13.4	8.8	8.9	8.9	9.0
Handan	17.1	17.2	17.2	17.7	15.3	15.4	15.5	15.7	12.6	12.7	12.8	13.2
Xingtai	15.6	15.7	15.9	16.3	14.0	14.1	14.2	14.5	11.6	11.7	11.8	12.3
Baoding	23.6	23.8	24.2	24.9	20.9	21.1	21.4	22.0	17.7	17.9	18.3	19.0
Zhangjiakou	13.7	13.8	13.9	13.9	13.7	13.8	13.9	13.9	9.17	9.3	9.4	9.4
Chengde	17.7	17.8	17.8	17.9	17.7	17.8	17.8	17.9	11.9	11.9	12.0	12.0
Cangzhou	18.3	18.6	22.4	22.9	15.9	16.2	18.3	18.7	14.0	14.2	18.1	18.5
Langfang	12.6	12.8	13.1	13.7	11.3	11.5	11.8	12.2	9.5	9.7	9.9	10.5
Hengshui	9.3	9.3	12.9	13.1	7.7	7.8	9.6	9.8	7.2	7.3	10.9	11.0
BTH	265.8	284.6	302.4	317.7	248.6	262.1	274.8	286.8	193.3	212.0	229.8	245.0

**Table 2:** Urbanization development under different scenarios in BTH urban agglomeration.

Cities	Time	Core Development Mode			Subcore Development Mode			Multinode Development Mode		
		*TP* (million)	*UR* (%)	*GDP* (billion yuan)	*TP* (million)	*UR* (%)	*GDP* (billion yuan)	*TP* (million)	*UR* (%)	*GDP* (billion yuan)
Beijing	2020	23.6	89.5	1985	23.1	88.5	1988	23.1	87.5	1982
	2025	25.4	92.5	2868	24.6	90.5	2852	24.2	89.5	2824
	2030	27.2	94.5	4089	26.5	92.5	4022	25.5	90.5	3941
Tianjin	2020	17.7	84.6	1919	18.0	85.6	1945	17.8	83.6	1909
	2025	19.9	86.6	2803	20.2	87.1	2938	20.2	84.1	2743
	2030	22.1	87.6	4120	22.4	89.1	4458	22.6	85.1	3929
Shijiazhuang	2020	11.5	61.3	701	11.7	63.3	708	11.6	62.3	702
	2025	12.2	66.3	976	12.6	69.3	1004	12.5	67.3	975
	2030	13.0	74.3	1345	13.4	74.3	1410	13.5	72.3	1346
Tangshan	2020	7.9	60.3	637	7.9	62.3	640	8.0	61.3	633
	2025	8.2	62.3	850	8.2	64.3	867	8.3	63.3	830
	2030	8.4	64.3	1132	8.4	66.3	1173	8.7	65.3	1087
Qinhuangdao	2020	3.3	61.1	188	3.3	62.1	189	3.3	63.1	190
	2025	3.5	66.1	253	3.5	67.1	258	3.5	68.1	262
	2030	3.6	70.1	334	3.6	71.1	346	3.7	72.1	356
Handan	2020	9.2	54.0	419	9.2	55.0	422	9.2	56.0	424
	2025	9.3	58.0	565	9.3	60.0	580	9.4	62.0	586
	2030	9.5	62.0	750	9.5	64.0	787	9.6	66.0	800
Xingtai	2020	7.1	54.0	227	7.1	52.0	233	7.1	53.0	233
	2025	7.2	58.0	296	7.2	57.0	306	7.2	58.0	310
	2030	7.2	62.0	381	7.2	62.0	398	7.3	63.0	408
Baoding	2020	12.7	56.7	510	12.7	60.7	515	12.7	58.7	518
	2025	13.3	60.7	717	13.3	64.7	742	13.4	62.7	735
	2030	13.9	64.7	996	13.9	66.7	1060	14.1	65.7	1035
Zhangjiakou	2020	4.8	56.2	163	4.8	58.2	163	4.8	57.2	174
	2025	4.8	60.2	227	4.8	62.2	228	4.9	61.2	244
	2030	4.90	62.2	318	4.9	64.2	322	5.0	63.2	339
Chengde	2020	3.9	53.0	134	3.9	55.0	135	4.0	57.0	143
	2025	4.0	58.0	192	4.0	60.0	195	4.1	61.0	208
	2030	4.1	61.0	272	4.1	62.0	282	4.3	63.0	302
Cangzhou	2020	8.0	54.2	373	8.0	56.2	376	8.0	55.2	382
	2025	8.3	59.2	529	8.3	61.2	546	8.5	60.2	547
	2030	8.7	63.2	738	8.7	65.2	781	9.0	64.2	772
Langfang	2020	4.9	60.0	271	4.9	62.0	273	4.9	61.0	275
	2025	5.1	65.0	392	5.2	67.0	403	5.3	66.0	395
	2030	5.4	68.0	567	5.7	70.0	593	5.7	69.0	564
Hengshui	2020	4.4	54.3	179	4.4	56.3	183	4.4	58.3	187
	2025	4.5	58.3	245	4.5	60.3	256	4.5	62.3	265
	2030	4.5	60.3	330	4.5	62.3	356	4.6	64.3	375
BTH	2020	118.9	67.5	7707	118.9	68.6	7769	118.9	67.8	7752
	2025	125.7	71.6	10913	125.8	72.5	11176	126.0	71.5	10924
	2030	132.6	75.0	15371	132.8	75.7	15986	133.6	74.2	15255

Note: *TP*: total population; *UR*: urbanization rate; *GDP*: gross domestic product.
